# Genetic diversity of *κ*-casein (*CSN3*) and lactoferrin (*LTF*) genes in the endangered Turkish donkey (*Equus asinus*) populations

**DOI:** 10.5194/aab-62-77-2019

**Published:** 2019-02-25

**Authors:** Fulya Özdil, Hasan Bulut, Raziye Işık

**Affiliations:** Namık Kemal University, Department of Agricultural Biotechnology, 59030 Tekirdağ, Turkey

## Abstract

In this study, the κ-casein (*CSN3*) and lactoferrin (*LTF*) genes which were found in association with milk production traits in different animal species were studied firstly in Turkish donkey populations. A total of 108 donkeys from different regions of Turkey were used in order to reveal the different genotypes of *CSN3* and *LTF* genes by using polymerase chain reaction – restriction fragment length polymorphism (PCR-RFLP) and DNA sequencing methods. To determine the genetic polymorphism, we attempted to digest a fragment of 235 bp of the *CSN3* gene and a fragment of 751 bp of the *LTF* gene using *Pst*I, and *Dra*II, *Eag*I and *Mbo*I restriction enzymes, respectively. Neither the *CSN3* gene nor the *LTF* gene had enzyme recognition sites with the *Pst*I, *Dra*II and *Mbo*I restriction enzymes in all of the studied samples. However, the *LTF* gene was only distinguished with the *Eag*I restriction enzyme. Three genotypes were identified in the *LTF* gene with the *Eag*I restriction enzyme: GG homozygotes (667, 84 bp), AG heterozygotes (751; 667, 84 bp) and AA homozygotes (751 bp). The transition from guanine to adenine in 89 bp of the *LTF* gene lacks the restriction site and different genotypes are obtained. This novel single nucleotide polymorphism (SNP) has been firstly detected in donkeys. According to the results, the G allele was predominant in the *LTF-Eag*I gene in the studied Turkish donkey populations. In this study, all the genotype distributions of *LTF-Eag*I were not found in Hardy–Weinberg equilibrium (P<0.05). The *CSN3* and *LTF* genes have not been studied before in donkeys, so the results are the preliminary results of these gene regions in donkeys.

## 1 Introduction

Donkeys belong to the order of odd-toed ungulates (*Perissodactyla*) and sub-order of Horse-like (*Hippomorpha*) and the horse family (*Equidae*). This family includes the genus horse (*Equus*). Currently, *Equus *is represented by eight extant species: domestic horse (*E. caballus*), Przewalski's horse (*E. przewalskii*), kiang (*E. kiang*), Asiatic wild ass (*E. hemionus*), African wild ass (*E. africanus*), mountain zebra (*E. zebra*), plains zebra (*E. quagga*) and Grévy's zebra (*E. grevyi*) (Moehlman, 2002; Kugler et al., 2008). The ancestors of the domestic donkey (*Equus asinus*) are the African wild asses. They were divided into three subspecies: north African wild ass (*Equus asinus atlanticus*), Nubian wild ass (*Equus asinus africanus*) and Somali wild ass (*Equus asinus somalicus*). *Equus asinus atlanticus* was already extinct in Roman times. The Nubian wild ass, from which our domestic donkey (*Equus asinus asinus*) mainly descends, is also threatened by extinction (Rubenstein, 2001).

**Figure 1:**
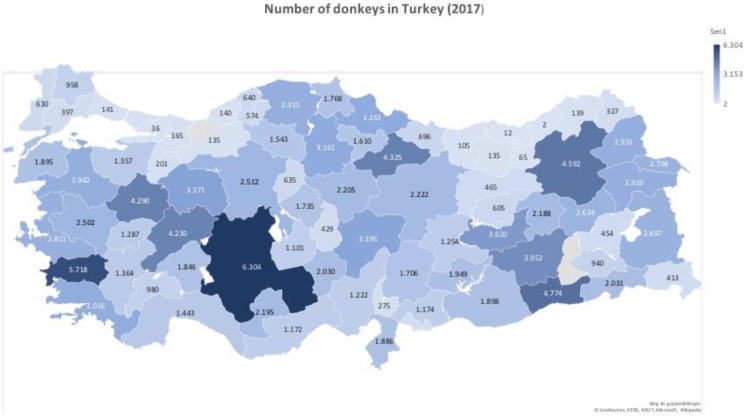
The sampling locations and the sample sizes of donkeys used in this study (©GeoNames, Microsoft, Navteq, Wikipedia).

Draught animals, such as horses, mules, donkeys and so on, play a key role in the economy of developing countries by being the main source of power in transport and traction. But along with the industrialization, when motor power became available and affordable, people started to replace these animals with machines. So, in many countries, donkey breeds which were used as pack animals in rural areas have become extinct or critically endangered (Kugler et al., 2008; Lauvie et al., 2011). In the last years, donkey populations have declined dramatically in Turkey. The donkey population of Turkey has declined 86 % between the years 1990 and 2016: 1 084 000 to 155 158 heads, respectively (Anonymous, 2018). The number of donkeys in different provinces of Turkey is given on the map of Turkey (Fig. 1).

To date, little information has been found about morphologic (Yılmaz and Ertuğrul, 2012; Yılmaz and Wilson, 2013; Yalçın, 2016) and genetic characteristics (Kul et al., 2016; Yalçın, 2016; Yatkın, 2019) of Turkish donkey breeds, even if the donkey breeds of Turkey have not been clarified particularly. According to FAO, Domestic Animal Diversity Information System (DAD-IS), Turkey has three native donkey breeds: Anatolian, Merzifon and Karakaçan donkey breeds. But no morphologic or genetic information is found in this database.

All over the world, donkeys which are under threat of extinction have to be characterized both morphologically and genetically in order to constitute conservation strategies. For the last years, donkey milk has widely used to overcome some diseases such as asthma and cancer. Due to its rich content, the scientific interest in donkey milk has been increased recently (Salimei et al., 2004; Piccione et al., 2008). So, donkey milk composition and its protein content have become important and have to be characterized in different breeds. Casein and lactoferrin genes, which are found in association with milk production traits in cattle and sheep, should be investigated in donkeys in order to identify the gene regulation of donkey milk genetic parameters. So, the aim of this study is to search and introduce the genetic variation in terms of κ-casein and lactoferrin genes in donkey populations reared in different provinces of Turkey. In addition to this, the genetic characterization of casein and lactoferrin genes in donkeys is conducted for the first time in Turkey.

## 2 Materials and methods

### 2.1 Sampling and DNA isolation

**Figure 2:**
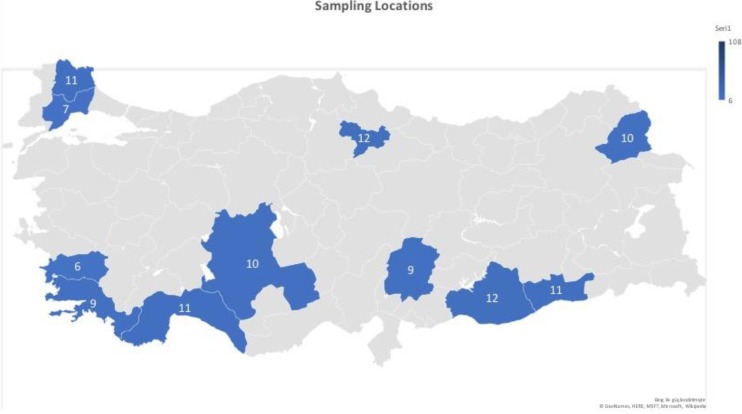
The sampling locations and the sample sizes of donkeys used in this study. ©GeoNames, Microsoft, Navteq, Wikipedia (the Turkey map is created with Microsoft Excel enhanced via Bing).

In this study, we tried to make a Turkey survey in terms of donkey distributions while collecting samples. For this purpose, 10 ml blood samples were collected from the jugular vein of 108 donkeys in 11 different provinces including Kırklareli (11), Tekirdağ (7), Aydın (6), Muğla (9), Antalya (11), Konya (10), Amasya (12), Kahramanmaraş (9), Şanlıurfa (12), Mardin (11) and Kars (10) (Fig. 2). Blood samples were stored in vacuum tubes containing K3 ethylenediaminetetraacetic acid (EDTA) as anticoagulant. Genomic DNA was isolated using fenol–chloroform extraction from blood samples.

### 2.2 PCR-RFLP and DNA sequencing

A total of 751 bp of the lactoferrin (*LTF*) gene was amplified by using polymerase chain reaction (PCR) via newly designed primers given below.

 F: 5'-GCAACAACGAGAATGAGAACAAGT-3'
 R: 5'-ATTTCACCACCAGATGGCGT-3-'

The given primers were designed from the whole genome shotgun sequence of *Equus asinus* isolate Maral har breed Guanzhong donkey's National Center for Biotechnology Information (NCBI) reference sequence (NW_014636647).

The PCR reaction mix of the *LTF* gene is as follows: 25 µL of PCR volume contained 100 ng of genomic DNA, 1×PCR buffer, 0.5 µM of each primer, 200 µM dNTP, 1.5 mM ${\mathrm{MgCl}}_{2}$ and 2.5 units of Taq DNA polymerase (Invitrogen^™^, Life Technologies). The PCR cycling protocol was 4 min at 94 ${}^{\circ }$C, 35 cycles of 95 ${}^{\circ }$C for 30 s, 68 ${}^{\circ }$C annealing for 1 min, 74 ${}^{\circ }$C for 1 min, with a final extension of 74 ${}^{\circ }$C for 10 min.

A total of 235 bp of the κ-casein gene (*CSN3*) was amplified using the given primers below with slight modifications (Selvaggi and Dario, 2011).

 F: 5'-GATGACAACTCTATTTCCCCCT-3'
 R: 5'-CCAGGGTCAGGTCTTGCT-3'

The PCR reaction mix of the *CSN3 *gene is as follows: 25 µL of PCR volume contained 100 ng genomic DNA, 1×PCR buffer, 0.5 µM of each primer, 200 µM dNTP, 2 mM ${\mathrm{MgCl}}_{2}$ and 1 unit of Taq DNA polymerase (Invitrogen^™^, Life Technologies). The PCR cycling protocol was 4 min at 95 ${}^{\circ }$C, 35 cycles of 95 ${}^{\circ }$C for 1 min, 63 ${}^{\circ }$C annealing for 30 s, 74 ${}^{\circ }$C for 1 min, with a final extension at 74 ${}^{\circ }$C for 10 min.

**Figure 3:**
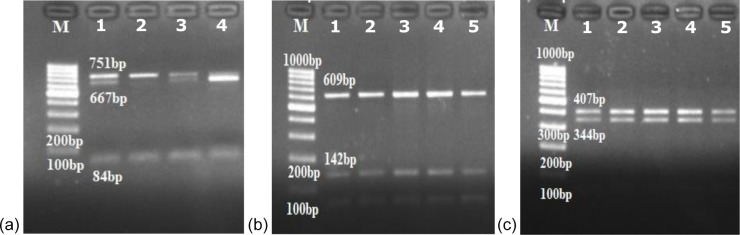
*Eag*I, *Mbo*I and *Dra*II restriction patterns of the *LTF* gene in Turkish donkey populations. **(a)** *Eag*I digestion of the *LTF* gene segment, lanes 1 and 3: AG heterozygotes (751; 667, 84 bp); lane 2: AA homozygote (751 bp); lane 4: GG homozygote (667, 84 bp); **(b)** *Mbo*I digestion of the *LTF* gene segment (609, 142 bp); **(c)** *Dra*II (*Eco*0109I) digestion of the *LTF* gene segment (407, 344 bp); M – DNA size marker (Invitrogen^™^ 100 bp DNA ladder).

**Figure 4:**
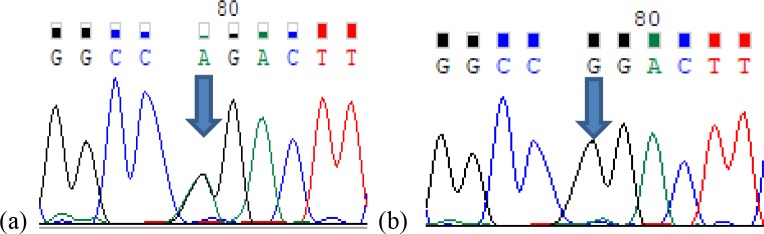
Partial sequence of the *LTF* gene showing the G → A transition. **(a)** *Eag*I heterozygote genotype (AG); **(b)** homozygote genotype (GG).

A total of 751 bp of the *LTF* gene was digested with *Dra*II (*Eco*0109I), *Mbo*I and *Eag*I restriction enzymes (ER0261, ER0811, ER0331, Thermo Fisher Scientific) at 37 ${}^{\circ }$C for 16 h and inactivated at 65 ${}^{\circ }$C for 20 min. In total, 235 bp of the *CSN3 *gene was digested with *Pst*I restriction enzyme (ER0611, Thermo Fisher Scientific) at 37 ${}^{\circ }$C for 16 h. PCR products and restriction fragments were electrophoresed on 2.5 % agarose gel stained with SafeView^™^ Classic (Applied Biological Material Inc., Canada) and photographed in the Vilber Lourmat gel imaging system. The allele frequencies of the *LTF* gene via *Eag*I restriction enzyme were calculated using the POPGENE software. The Hardy–Weinberg equilibrium was also checked using the same software (Yeh et al., 1999).

Also different *LTF *genotypes that were obtained via *Eag*I digestion were sequenced on an Applied Biosystems 3500XL genetic analyzer system (Applied Biosystems, USA) in order to verify the sequence variations of the *Eag*I restriction site. We sequenced two samples each from 11 locations in Turkey. Sequences were aligned with BioEdit Sequence Alignment Editor by using Clustal W multiple alignment modules (Hall, 2011). The sequences of *LTF* fragments were analyzed using the MEGA 7 (Molecular Evolutionary Genetic Analysis, version 6.0) software (Tamura et al., 2013).

## 3 Results

In this study, we conducted the polymerase chain reaction – restriction fragment length polymorphism (PCR-RFLP) method in order to find out variations in κ-casein and lactoferrin genes. The *LTF* gene was digested with *Dra*II, *Mbo*I and *Eag*I restriction enzymes, whereas the *CSN3* gene was digested with the *Pst*I restriction enzyme in order to validate the genetic polymorphism in 11 donkey populations of Turkey (Fig. 2).

In the *LTF* gene, no genetic polymorphism was found with *Mbo*I and *Dra*II restriction enzymes. All the studied samples had one restriction site, the 609th position with *Mbo*I and 344th position with *Dra*II restriction enzymes which gave 609–142 and 407–344 bp fragments in *Mbo*I and *Dra*II digestion, respectively (Fig. 3). In the *CSN3* gene, there was no digestion with the *Pst*I restriction enzyme; only a 235 bp PCR product was obtained in all of the studied samples.

We found polymorphism with the *Eag*I restriction enzyme in the *LTF* gene. The *LTF* gene consists of 18 exons and 17 introns. The region that we have analyzed both with PCR-RFLP and DNA sequencing includes the partial 14th exon and 14th intron. Most of the samples had the *Eag*I restriction site in the 14th intron of this gene and this restriction gave 667 and 84 bp fragments. The transition from guanine to adenine (G → A) in 89 bp of the *LTF* gene in the 14th intron lacks this restriction site and no digestion is found. Therefore, three genotypes were identified in the *LTF* gene with the *Eag*I restriction enzyme (Fig. 3a): GG homozygotes (667, 84 bp), AG heterozygotes (751; 667, 84 bp) and AA homozygotes (751 bp) in Turkish donkey populations.

The genotype and allele frequencies of *LTF* gene *Eag*I variants are listed in Table 1. AG heterozygotes were found high in all of the 11 sampling locations (60.2 %) except for Kars. GG homozygotes were found high only in the Kars province (80 %). On the other hand, AA and GG homozygotes were not found in the Kahramanmaraş and Muğla provinces, respectively. The *LTF*-A allele frequency was 0.45, whereas the *LTF*-G allele frequency was 0.55. According to the results, G allele was predominant in the *LTF-Eag*I gene in the studied Turkish donkey populations. In this study, all the genotype distributions of *LTF-Eag*I were not found in Hardy–Weinberg equilibrium (P<0.05).

**Table 1:** The genetic and allelic frequencies of *LTF-Eag*I in donkeys.

Locus		*LTF* genotypes			*LTF* allele		χ2
					Frequency		
		AA	AG	GG	A	G	
	N	16	65	27			
*LTF*	Obs.	14.8	60.2	25.0	0.45	0.55	5.6
	Exp.	21.9	53.5	32.6			

In addition, according to the sequence results of the *LTF* gene, we have determined a novel G insertion at 91st position of the gene in all of the studied samples when we blast our sequences with the whole genome shotgun sequence of *Equus asinus* isolate Maral har breed Guanzhong donkey's NCBI reference sequence (NW_014636647).

## 4 Discussion

Around the middle of the 20th century, as a consequence of industrialization in agriculture and the spreading several highly selected breeds, many animal populations have become extinct or are declining and endangered. In many countries, donkey breeds which were used as pack animals in rural areas have become extinct or critically endangered. In the last years, donkey populations have declined dramatically in Turkey. The donkey population of Turkey has declined 86 % between the years 1990 and 2016: 1 084 000 to 155 158 heads respectively (Anonymous, 2018). The number of donkeys in different provinces of Turkey is given on the map of Turkey (Fig. 1). It is seen that in some of the provinces almost all the donkey populations have disappeared. So, both morphological and genetic studies have to be conducted on Turkish native donkey breeds.

In this study, we have identified lactoferrin and casein genes in donkeys which are found in association with milk traits in farm animals. Lactoferrin and casein genes are studied firstly in Turkish native donkey populations. We used PCR-RFLP and DNA sequencing methods in order to find out different genotypes in these gene regions of Turkey's donkeys. In the *LTF* gene, we did not distinguish any genetic polymorphism with *Mbo*I and *Dra*II restriction enzymes, whereas polymorphism was found with the *Eag*I restriction enzyme in the *LTF* gene. The lactoferrin gene has not been studied before in donkeys, so these results are the preliminary results of this gene region in donkeys.

In this study, we also identified the κ-casein gene (*CSN3*) in Turkish native donkey populations. Although lots of studies are found in association between *CSN3* gene polymorphism and economic quantitative traits in other species, very few studies were conducted in this gene region in equine species. Selvaggi et al. (2015) analyzed exon 1 of the κ-casein gene in four Italian horse populations and Martina Franca donkeys. In their study, *Pst*I digestion in the *CSN3* gene was performed to discover the presence of c.-66A > G polymorphism. This gene region was found to be polymorphic in horses, but similar to our results, no genetic variability was observed in Martina Franca donkey breed.

This study provides insight on genetic studies of donkey breeds; κ-casein (*CSN3*) and lactoferrin (*LTF*) genes are firstly studied in Turkish donkey populations. Further characterization is needed to analyze donkey populations in order to find out new polymorphisms, and mitochondrial studies have to be conducted to identify the donkey breeds of Turkey. Also the possible relationships between the single nucleotide polymorphisms (SNPs) and some milk performance traits should be determined in donkeys.

## Ethical statement

Tekirdağ Namık Kemal University, Animal Experiments Local Ethics Committee's approval has been obtained for project TOVAG-215O555 on 3 September 2015 and used for this study.

## Data Availability

The data sets are available upon request from the corresponding author.
